# Circ‐POSTN promotes the progression and reduces radiosensitivity in esophageal cancer by regulating the miR‐876‐5p/FYN axis

**DOI:** 10.1111/1759-7714.15273

**Published:** 2024-03-29

**Authors:** Alan Chu, Chen Sun, Zongwen Liu, Shijia Liu, Mengxi Li, Rui Song, Lanlan Gan, Yongtai Wang, Ruitai Fan

**Affiliations:** ^1^ Department of Radiation Oncology The First Affiliated Hospital of Zhengzhou University Zhengzhou Henan China; ^2^ Department of Radiation Oncology The Second Affiliated Hospital of Zhengzhou University Zhengzhou Henan China

**Keywords:** circ‐POSTN, esophageal cancer, FYN, miR‐876‐5p, proliferation

## Abstract

**Background:**

Circular RNAs (circRNAs) play critical roles in the tumorigenesis and radiosensitivity of multiple cancers. Nevertheless, the biological functions of circRNA periostin (circ‐POSTN) in esophageal cancer (EC) progression and radiosensitivity have not been well elucidated.

**Methods:**

The expression of circ‐POSTN, microRNA‐876‐5p (miR‐876‐5p), and proto‐oncogene tyrosine‐protein kinase (FYN) was analyzed by quantitative reverse transcription PCR (RT‐qPCR). Cell proliferation was assessed by MTT, colony formation, and 5‐ethynyl‐2′‐deoxyuridine (EDU) assays. All protein levels were detected by western blot assay. Cell apoptosis and invasion were assessed by flow cytometry analysis and transwell assay, respectively. Dual‐luciferase reporter and RNA immunoprecipitation (RIP) assays were used to validate the interaction between miR‐876‐5p and circ‐POSTN or FYN. The role of circ‐POSTN in vivo was explored by establishing mice xenograft model.

**Results:**

Circ‐POSTN was overexpressed in EC tissues and cells. Knockdown of circ‐POSTN inhibited cell proliferation and invasion and elevated apoptosis and radiosensitivity in EC cells. MiR‐876‐5p was a direct target of circ‐POSTN, and its knockdown reversed the role of sh‐circ‐POSTN in EC cells. FYN was a direct target of miR‐876‐5p, and FYN elevation weakened the effects of miR‐876‐5p overexpression on the progression and radiosensitivity of EC cells. Moreover, circ‐POSTN acted as a miR‐876‐5p sponge to regulate FYN expression. Circ‐POSTN interference also suppressed tumor growth and enhanced radiosensitivity in vivo.

**Conclusion:**

Circ‐POSTN knockdown inhibited proliferation and invasion, but increased apoptosis and enhanced radiosensitivity in EC cells via modulating miR‐876‐5p/FYN axis, which might be a potential diagnostic and therapeutic target for EC.

## Graphical abstract

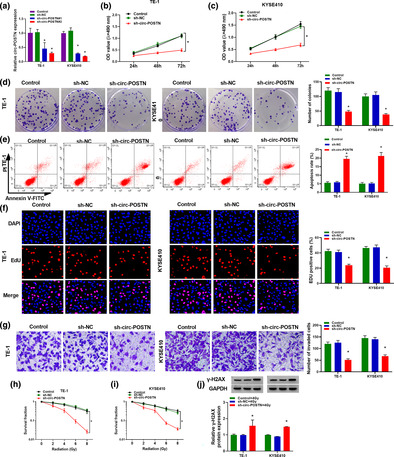

## INTRODUCTION

Esophageal cancer (EC), with a high incidence worldwide, is one of the most frequent gastrointestinal malignancies.^1^ Despite systemic treatments markedly improving patient survival in recent years, the prognosis of patients is still poor.^2^ Radiation therapy is still an important clinical treatment of EC, but radioresistance is a significant clinical hindrance to EC therapy.^3^, ^4^ Hence, clarifying the underlying mechanisms of EC is important to discover novel effective therapeutic strategies and enhance radiosensitivity.

Circular RNAs (circRNAs), with a covalently closed loop structure, are a large class of primarily noncoding RNAs (ncRNAs).^5^, ^6^ Recently, emerging evidence has proved that circRNAs play crucial roles in tumorigenesis and radiosensitivity of EC. For example, circRNA has_circ_0007380 was highly expressed in EC and promoted EC cell proliferation and decreased radiosensitivity.^7^ Moreover, circRNA_0001686 and circRNA_0000554 were upregulated in radioresistant EC cell lines.^8^, ^9^ CircRNA periostin (circ‐POSTN; also known as hsa_circ_0030018, chr13:38136718–38 161 065) is derived from the POSTN gene and has been reported to facilitate EC cell proliferation, migration, and epithelial‐mesenchymal transition (EMT) process.^10^ However, the function of radiosensitivity of circ‐POSTN and its relative molecular mechanism remains largely unknown in EC.

CircRNAs usually exert their functions through functioning as microRNA (miRNA) sponges.^11^ MiRNAs are a class of short ncRNAs with about 22 nucleotides in length that can affect gene expression by mediating mRNA degradation or inhibiting mRNA translation.^12^ MiR‐876‐5p has been suggested to function as a tumor‐inhibiting factor in cancers.^13^, ^14^, ^15^ In addition, elevation of miR‐876‐5p has been reported to inhibit EC progression.^8^, ^16^ Nevertheless, the function of miR‐876‐5p on radiosensitivity in EC has not previously been reported.

Proto‐oncogene tyrosine‐protein kinase (FYN) belongs to the SRC family of kinases (SFKs) and acts as an oncogene in multiple cancers.^17^, ^18^ Furthermore, it has been demonstrated that high expression of FYN in EC promoted EC tumorigenesis.^19^, ^20^ However, the detailed function of FYN in EC is unclear.

In recent years, increasing research has focused on the circRNA‐miRNA‐mRNA regulatory mechanism that circRNAs might derepress target mRNA expression via competitively binding to miRNAs. In the current paper, bioinformatic analysis found that miR‐876‐5p possessed some binding sites with circ‐POSTN or FYN. Therefore, in the present study, we aimed to validate the role of circ‐POSTN in the progression and radiosensitivity of EC, and to illuminate whether the involvement of circ‐POSTN in EC function was mediated by miR‐876‐5p/FYN axis.

## METHODS

### Clinical specimens

Thirty resected EC tissue samples and paired normal tissue specimens were harvested from EC patients at the second affiliated hospital of Zhengzhou University. After being collected, these specimens were timely snap‐frozen in liquid nitrogen until RNA or protein extraction. Each patient signed their informed consent. This study was authorized by the Research Ethics Committee of the second affiliated hospital of Zhengzhou University. The detailed clinical characteristics of patients are described in Table 1.

**TABLE 1:** Relationship between circ‐POSTN expression and clinicopathological features of esophageal cancer patients.

	Characteristics *n* = 30	Circ‐POSTN expression		*p*‐value^a^
		Low (*n* = 15)	High (*n* = 15)	
Age (years)				0.7152
≤50	14	8	6	
>50	16	7	9	
TNM grade				0.0253*
I + II	13	10	3	
III	17	5	12	
Lymph node metastasis				0.0209*
Positive	19	6	13	
Negative	11	9	2	
Tumor size				0.0092*
≤5 cm	16	12	4	
>5 cm	14	3	11	

Abbreviation: TNM, tumor‐node‐metastasis.

* *p* < 0.05.

^a^ Chi‐square test.

### Cell culture and transfection

Human esophageal epithelial cell line (HEEC) and EC cell lines (KYSE510 and ECA109) were purchased from BeNa Culture Collection (Beijing, China). EC cell lines (TE‐1 and KYSE410) were obtained from Procell. These cells were cultivated in Dulbecco's modified Eagle medium (DMEM) or RPMI‐1640 medium (Invitrogen) that consisted of 10% fetal bovine serum (FBS; Invitrogen) in a moist incubator with 5% CO_2_ at 37°C.

The short hairpin RNAs (shRNAs) targeting circ‐POSTN (sh‐circ‐POSTN#1 and sh‐circ‐POSTN#2), miR‐876‐5p mimic or inhibitor (miR‐876‐5p or anti‐miR‐876‐5p), circ‐POSTN overexpression vector, FYN overexpression vector (FYN) and matched controls (sh‐NC, miR‐NC, anti‐miR‐NC, pCD5‐ciR empty vector or pcDNA empty vector) were procured from RiboBio. Transfection of oligonucleotides and vector (50 nM siRNA, 50 nM mimics or inhibitors, 4 μg vector) was accomplished using lipofectamine 3000 (Invitrogen).

### Quantitative reverse transcription PCR (RT‐qPCR)

A TRIzol kit (Invitrogen) was employed to isolate total RNA following the manufacturer's instructions. For detecting gene expression, the cDNA was obtained using PrimeScript RT reagent Kit (TaKaRa). Then, the SYBR Green PCR kit (TaKaRa) with specific primers was employed for RT‐qPCR analysis on Bio‐Rad CFX96 system (Bio‐Rad). The 2^−ΔΔCt^ method was applied to analyze the expression of genes, followed by normalization to U6 (for miR‐876‐5p) or GAPDH (for circ‐POSTN, POSTN, and FYN). The primer sequences are listed in Table 2.

**TABLE 2:** The primer sequences for RT‐qPCR.

Name	Primer sequences (5'‐3')	
circ‐POSTN	Forward	5'‐GTTTGGACTTGGGAACAGGA‐3'
	Reverse	5'‐CACCATTTGTTGCAATCTGG‐3'
POSTN	Forward	5'‐GTGAGGAAGTTGCAAGCCAA‐3'
	Reverse	5'‐CACTGAGAACGACCTTCCCT‐3'
miR‐876‐5p	Forward	5'‐CGCGTGGATTTCTTTGTGAA‐3'
	Reverse	5'‐AACGAGACGACGACAGAC‐3'
FYN	Forward	5'‐TCTCCTTCTCCTTTCAGTCAGG‐3'
	Reverse	5'‐AGGTCCCCGTATGAGACGAA‐3'
GAPDH	Forward	5'‐AATGGGCAGCCGTTAGGAAA‐3'
	Reverse	5'‐GCGCCCAATACGACCAAATC‐3'
U6	Forward	5'‐CTCGCTTCGGCAGCACATATACT‐3'
	Reverse	5'‐ACGCTTCACGAATTTGCGTGTC‐3'

Abbreviations: FYN, proto‐oncogene tyrosine‐protein kinase; GAPDH, glyceraldehyde‐3‐phosphate dehydrogenase; POSTN, periostin; RT‐qPCR, reverse transcription‐quantitative polymerase chain reaction.

### RNase R treatment

To confirm the circularization of circ‐POSTN, total RNA was digested with or without RNase R (Epicenter). After incubation for 30 min at 37°C, the levels of circ‐POSTN and POSTN were explored by RT‐qPCR.

### Subcellular fractionation

The fractions of cytoplasm and nucleus were separated in TE‐1 and KYSE410 using the PARIS kit (Invitrogen). Next, RT‐qPCR was used for testing the expression of GAPDH, U6, and circ‐POSTN. U6 and GAPDH were used as controls.

### MTT assay

In summary, transfected TE‐1 and KYSE410 were plated in a 96‐well plate for 48 h, and then hatched with 3‐(4,5‐dimethylthiazol‐2‐yl)‐2,5‐diphenyl‐2H‐tetrazolium bromide (MTT) reagent (20 μL, 5 mg/mL, Beyotime) for 4 h. After that, dimethyl sulfoxide (DMSO; 200 μL, Beyotime) was placed in each well after removal of the cell culture medium. The absorbance at 490 nm was then disclosed by a microplate reader (Bio‐Rad).

### Irradiation (IR) and colony formation assay

Transfected TE‐1 and KYSE410 cells (or control cells) were plated into a six‐well plate. For IR treatment, cells were exposed to various doses of x‐ray radiation through a 6‐MV linear accelerator (Elekta). The dose rate was 6.0 Gy/min. X‐ray irradiation with a clinically calibrated irradiation field of 10 cm × 10 cm was performed at the Department of Radiation Oncology, Affiliated Hospital of Zhengzhou University (Henan). After that, TE‐1 and KYSE410 cells were grown for 14 days to form colonies. Next, the colonies were fixed with alcohol (75%, Sangon Biotech), and spotted with crystal violet (0.1%, Keygen Biotech). The survival fraction was computed as previously reported.^8^

### Flow cytometry

Transfected TE‐1 and KYSE410 cells were cultured, collected, and treated with Annexin V‐FITC and PI solution (Vazyme). Lastly, a flow cytometry (BD FACSCanto II, BD Biosciences) was utilized to examine cell apoptosis rate.

### 5‐ethynyl‐2′‐deoxyuridine (EdU) assay

Click‐iT EDU detection kit (YF 555, UElandy) was employed for cell proliferation analysis. Briefly, cells were maintained in 24‐well plates for 48 h, followed by hatching with EdU (50 μM). Then, cells were settled with paraformaldehyde (4%) and permeabilized with Triton‐X‐100 (0.5%), then spotted with DAPI solution. Images were photographed with a fluorescence microscope (DM4000B, Leica).

### In vitro invasion assay

Cell invasion ability was estimated in a 24‐well transwell chamber (Costar, Corning). In brief, transfected cells in medium (0.2 mL) with free serum were plated onto the superior chamber precoated with Matrigel (BD Biosciences). Meanwhile, the lower chamber was supplemented with 0.6 mL medium with 10% FBS. Then, cells were scraped out from the top chamber using a cotton swab following incubation for 24 h. Afterwards, cells that invaded the underside of the chamber were fixed by alcohol (75%) and then stained with crystal violet (0.1%) and recorded with a microscope (Leica) at a magnification of ×100.

### Western blot assay

RIPA lysis buffer (Keygen) was exploited to extract the total proteins. Extracted protein samples (about 40 μg) were separated using SDS‐PAGE (Beyotime). Afterward, proteins were transferred to nitrocellulose membranes (Millipore). After blockage with 5% milk (Beyotime) for 1–2 h, the membranes were probed with primary antibody against FYN (ab227304,1:2000, Abcam), γ‐H2AX (ab124781, 1:1000, Abcam), or GAPDH (ab37168, 1:3000, Abcam) at 4°C overnight, and secondary antibody (ab205718, 1:4000, Abcam) for 1 h. Enhanced chemiluminescence (ECL; Abcam) reagent was utilized to detect the protein signals. ImageJ software was applied to evaluate the density of bands, and the protein abundance was normalized by GAPDH.

### Dual‐luciferase reporter assay

The fragments of circ‐POSTN and FYN 3'UTR containing the predicated miR‐876‐5p binding sites were individually cloned into pmirGLO luciferase reporter vector (Promega) to create the wild‐type (WT) plasmids (WT‐circ‐POSTN, FYN 3'UTR‐WT). The sequence of predicted binding fragments was replaced to mutate the predicted binding sites of miR‐876‐5p in the circ‐POSTN and FYN 3'UTR, namely mutant (MUT) plasmids (MUT‐circ‐POSTN, FYN 3'UTR‐MUT). After that, TE‐1 and KYSE410 cells were introduced with luciferase reporter vectors and miR‐NC/miR‐876‐5p for 48 h and quantified using a dual‐luciferase reporter assay system (Promega).

### RNA immunoprecipitation (RIP) assay

EZ‐Magna RIP kit (Millipore) was employed for the RIP assay. In short, RIP lysis buffer was used to lyse TE‐1 and KYSE410 cells, and then cell lysates were hatched with magnetic beads conjugated with Ago2 antibody. Immunoglobulin G (IgG) antibody served as a negative control. The beads were washed with cold NT2 buffer and digested through proteinase K buffer to remove the nonspecific binding. The immunoprecipitated RNA was separated and RT‐qPCR for detecting circ‐POSTN, miR‐876‐5p, and FYN levels.

### In vivo tumor growth assay

Stable KYSE410 cell line expressing sh‐NC or sh‐circ‐POSTN were established by GenePharma and selected with puromycin for 4 weeks. Then stable KYSE410 cells (3 × 10^6^/mouse) were injected into the right back of BALB/c nude mice (6‐week‐old, male, Vital River). The sh‐NC or sh‐circ‐POSTN group were randomly divided into two groups after injection for 7 days (*n* = 5 for each group). One group functioned as a control and the other group was irradiated with 6 Gy x‐ray. X‐ray was used to irradiate the right back mass of nude mice. Tumor volume (V) was monitored every 4 days and calculated with the following formula: 0.5 × length×width^2^. Then, 27 days upon transplantation, the mice were euthanized. Excised tissues were removed for further testing. This assay was authorized by the committee of Animal Research of Life Science Ethics Review Committee of Zhengzhou University.

### Immunohistochemistry (IHC) analysis

Dissected tumors were settled in 10% (v/v) formaldehyde, paraffin‐embedded, and slid into 4 μm sections. Next, the sections were hatched with FYN (1:500, ab227304, Abcam), Ki67 (1:200, ab15580, Abcam) and MMP9 (1:250, ab76003, Abcam) for 12 h at 4°C, and secondary antibody (1:4000, ab205718, Abcam) for 1 h. These sections were then stained with diaminoaniline (DAB; Beyotime) and counterstained with hematoxylin (Beyotime). Lastly, the images were obtained using a microscope (×200, Leica).

### Statistical analysis

The data of at least three independent experiments are presented as the mean ± standard deviation. Difference was analyzed via student's *t*‐test (for two groups) or one‐way analysis of variance (ANOVA; for multiple groups) using GraphPad Prism 6.0. The correlation analysis between miR‐876‐5p and circ‐POSTN or FYN in EC tissues was performed using Spearman's rank correlation. *p*‐values less than 0.05 indicated statistical significance.

## RESULTS

### Circ‐POSTN was highly expressed in EC tissues and cells

**FIGURE 1:**
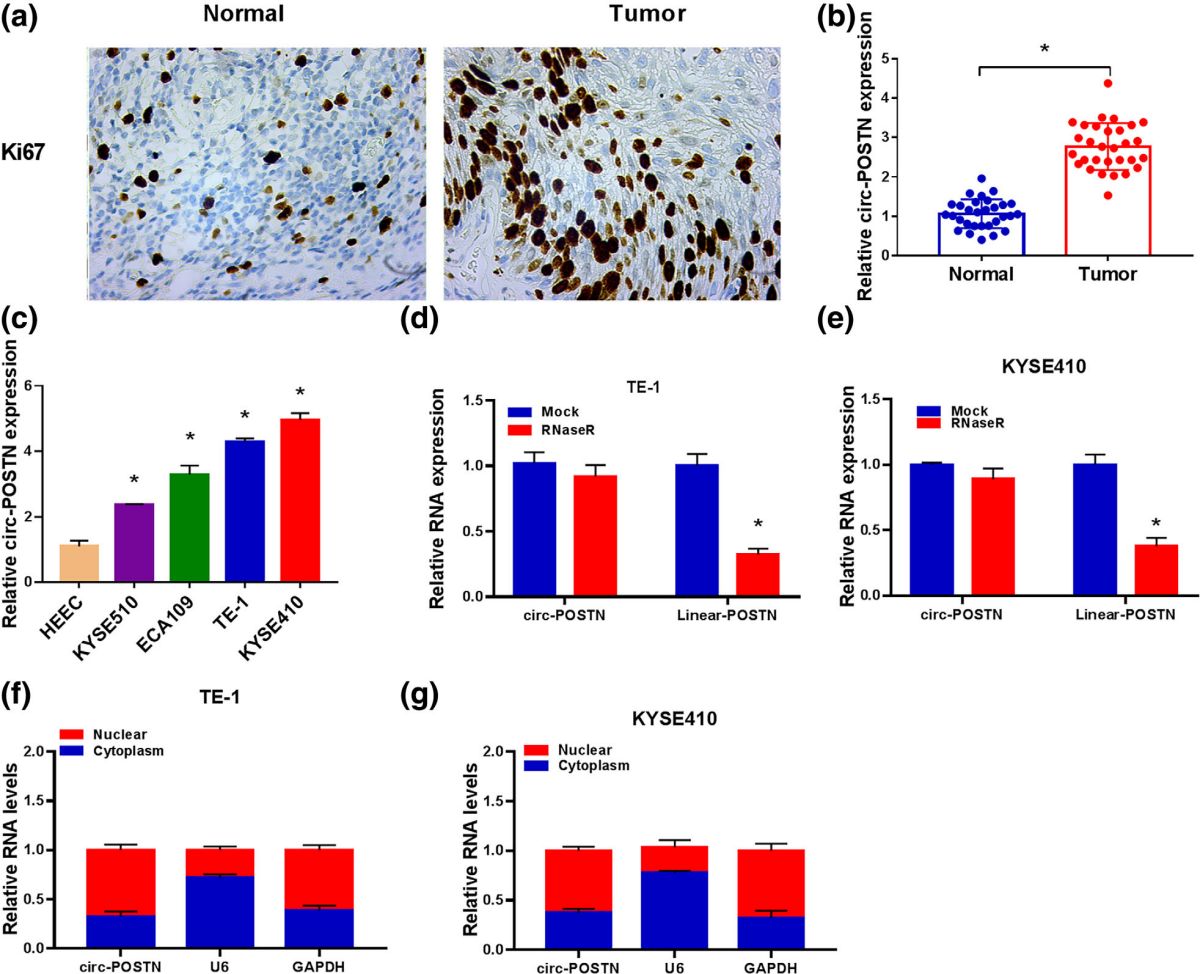
Circ‐POSTN expression was elevated in esophageal cancer (EC) tissues and cells. (a) Immunohistochemical (IHC) staining assay for Ki67 level in EC and normal tissues. (b) Relative circ‐POSTN expression was measured by reverse transcription‐quantitative polymerase chain reaction (RT‐qPCR) analysis in normal tissues (*n* = 30) and EC tissues (*n* = 30). (c) Circ‐POSTN expression in human esophageal epithelial cell line (HEEC) cells and EC cell lines (KYSE510, ECA109, TE‐1, and KYSE410) was detected by RT‐qPCR. (d and e) The expression levels of circ‐POSTN and POSTN were determined by RT‐qPCR in TE‐1 and KYSE410 cells after RNase R treatment. (F and G) Subcellular localization was performed to examine the distribution of circ‐POSTN in TE‐1 and KYSE410 cells *p < 0.05.

To explore the possible function of circ‐POSTN in EC, a total of 30 cases of EC tissues were collected. As shown in Figure 1a, the percentage of Ki67‐positive cells in EC tumor tissues was significantly increased compared to adjacent normal tissues. In addition, circ‐POSTN expression was highly expressed in EC tissues (*n* = 30) compared with that in normal tissues (*n* = 30) (Figure 1b). To probe the association of circ‐POSTN expression with clinicopathological features, the 30 patients with EC were then classified as shown in Table 1. The results showed that circ‐POSTN expression was associated with TNM grade, lymph node metastasis, and tumor size. We then further validated that the expression of circ‐POSTN was enhanced in EC cells (KYSE510, ECA109, TE‐1, and KYSE410) in contrast to that in HEECs (Figure 1c). To confirm the circularization of circ‐POSTN, total RNAs were treated with RNase R. The results revealed that circ‐POSTN was resistant to RNase R treatment, whereas the linear transcript POSTN was degraded by RNase R (Figure 1d,e). Subsequently, the localization of circ‐POSTN in TE‐1 and KYSE410 cells was explored. The data showed that circ‐POSTN was mainly located in the cytoplasm (Figure 1f,g). Hence, we speculated that circular RNA circ‐POSTN might serve as a ceRNA in EC.

### Silencing of circ‐POSTN repressed cell proliferation and invasion, but promoted apoptosis and enhanced radiosensitivity in EC cells

**FIGURE 2:**
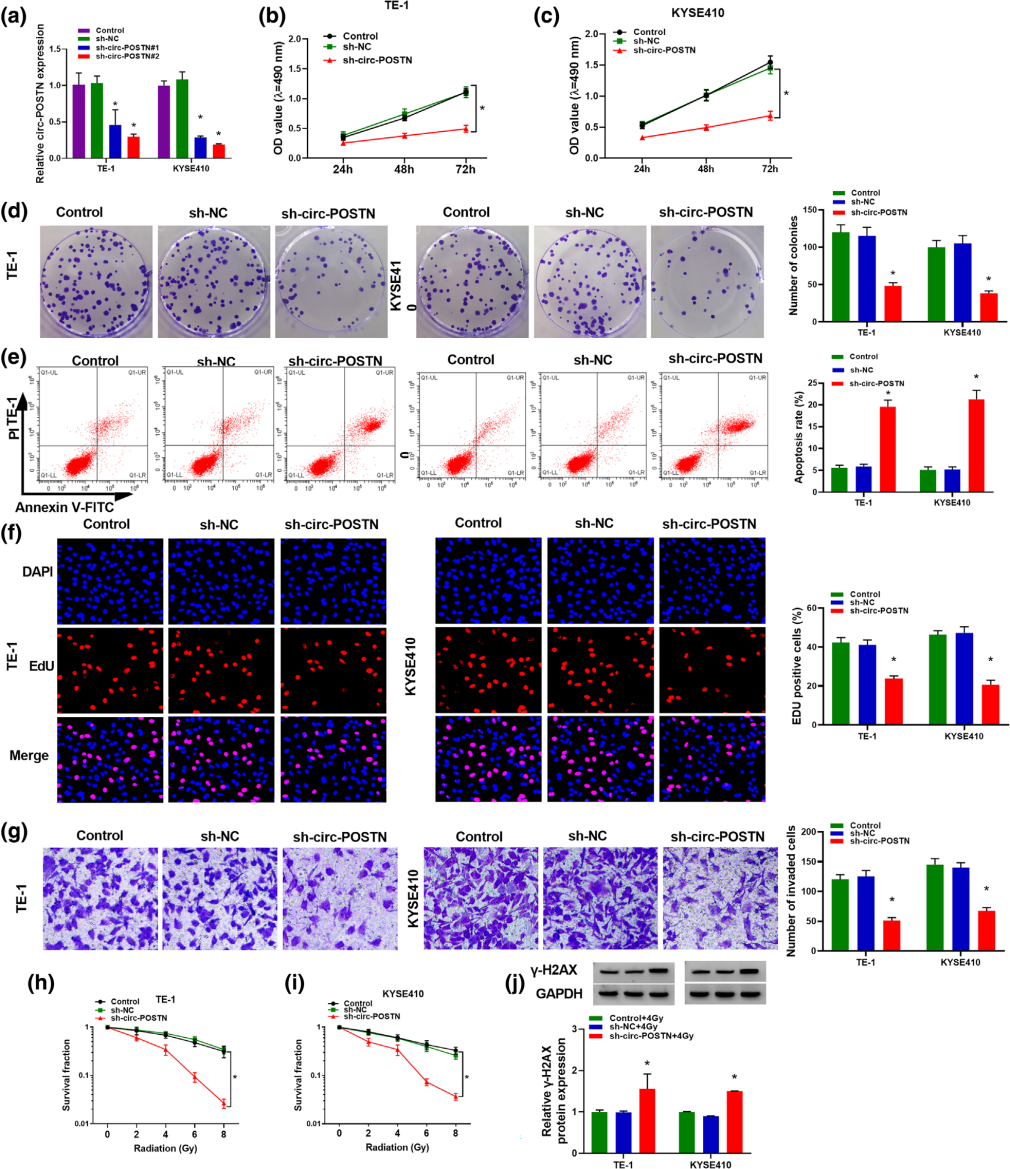
Circ‐POSTN silence inhibited cell proliferation and invasion, but promoted apoptosis and radiosensitivity of esophageal cancer (EC) cells. (a) The level of circ‐POSTN in untreated EC cells and EC cells transfected with sh‐NC, sh‐circ‐POSTN#1 or sh‐circ‐POSTN#2 was analyzed via reverse transcription‐quantitative polymerase chain reaction (RT‐qPCR) analysis. (b–j) TE‐1 and KYSE410 cells were divided into three groups: Control (untreated cells), sh‐NC, and sh‐circ‐POSTN group. (b and c) Cell viability of transfected EC cells was assessed by 3‐(4,5‐dimethylthiazol‐2‐yl)‐2,5‐diphenyl‐2H‐tetrazolium bromide (MTT) assay. (d) Cell colony‐formation ability was evaluated by colony formation assay. (e) Cell apoptosis was determined by flow cytometry analysis. (f) 5‐ethynyl‐2′‐deoxyuridine (EdU) assay was used to assess DNA synthesis. (g) Cell invasion was analyzed by transwell assay. (h and i) Colony formation assay was utilized to determine cell survival fraction in TE‐1 and KYSE410 cells exposed to radiation. (j) The protein level of γ‐H2AX was measured by western blot in TE‐1 and KYSE410 cells treated with 4 Gy radiation. *p < 0.05.

Next, the functional roles of circ‐POSTN in EC cells and radiosensitivity were explored by loss‐of‐function assays. As shown in Figure 2a, an obvious decrease of circ‐POSTN expression was observed in TE‐1 and KYSE410 cells transfected with sh‐circ‐POSTN#1 or sh‐circ‐POSTN#2, compared to the sh‐NC group or control group. Sh‐circ‐POSTN#2 also exhibited a more significant knockdown effect, which was chosen for subsequent research. MTT assay showed that circ‐POSTN interference remarkably suppressed the viability of TE‐1 and KYSE410 cells (Figure 2b,c). In addition, circ‐POSTN deficiency could reduce colony formation in TE‐1 and KYSE410 cells (Figure 2d). Flow cytometry showed that the knockdown of circ‐POSTN promoted TE‐1 and KYSE410 cell apoptosis (Figure 2e). EdU assay demonstrated that circ‐POSTN depletion inhibited cell proliferation in TE‐1 and KYSE410 cells, as EdU‐positive cells were reduced by circ‐POSTN knockdown (Figure 2f). Transwell invasion assay disclosed that cell invasion was evidently reduced in TE‐1 and KYSE410 cells with sh‐circ‐POSTN transfection in comparison with that in the control group or sh‐NC group (Figure 2g). Furthermore, the influence of circ‐POSTN on radiosensitivity was explored. The results manifested that circ‐POSTN knockdown apparently suppressed the survival fraction of TE‐1 and KYSE410 cells exposed to radiation (Figure 2h,i). In addition, the protein level of γ‐H2AX (a widely used marker for double‐strand DNA breaks) was significantly increased by circ‐POSTN knockdown in TE‐1 and KYSE410 cells after radiation treatment (Figure 2j). These results proved that circ‐POSTN silence repressed EC cell progression and improved radiosensitivity in vitro.

### Circ‐POSTN acted as a sponge for miR‐876‐5p

**FIGURE 3:**
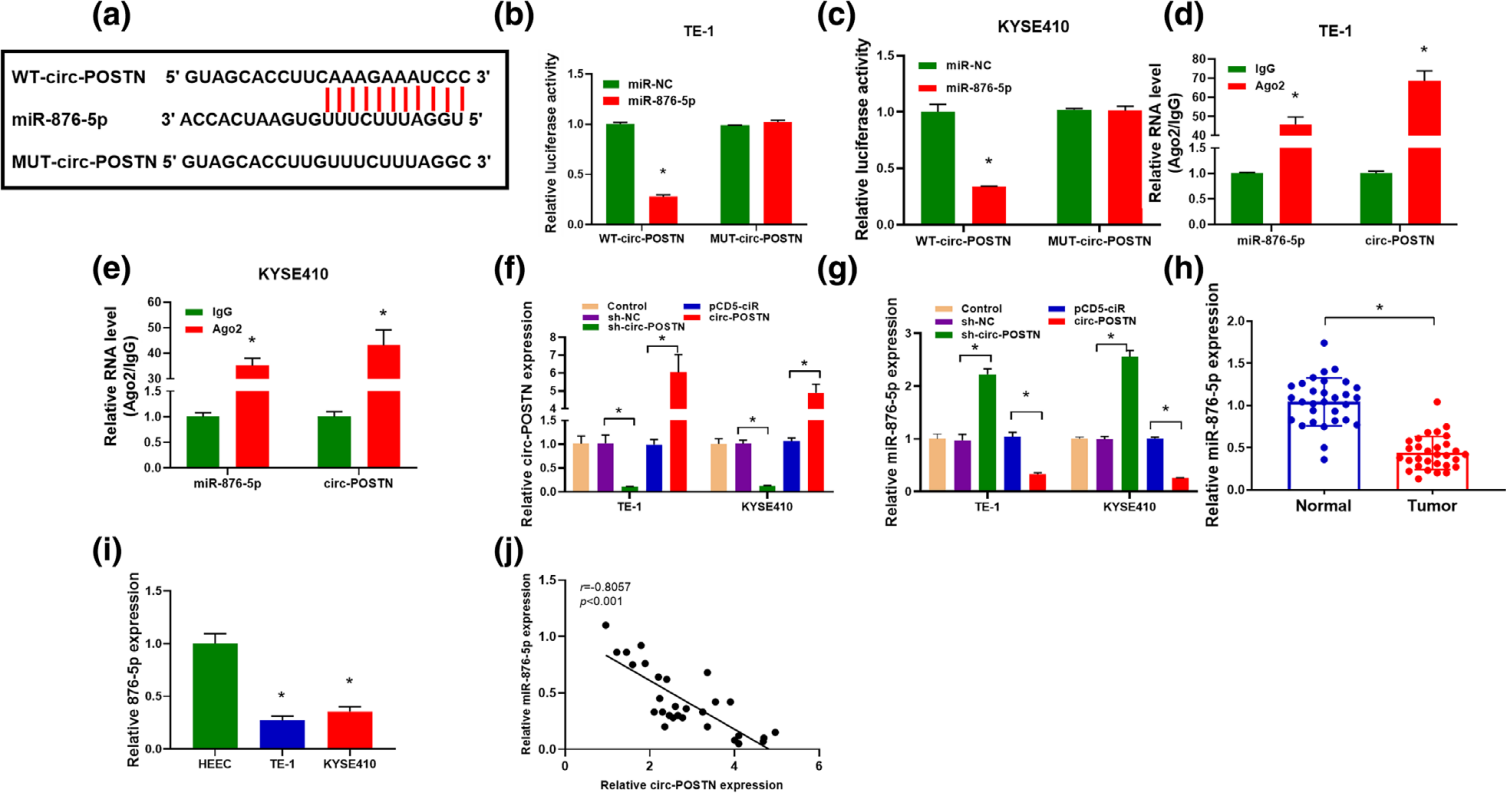
Circ‐POSTN functioned as a sponge for miR‐876‐5p. (a) The complementary binding sites between circ‐POSTN and miR‐876‐5p are shown. (b and c) The luciferase activity in TE‐1 and KYSE410 cells cotransfected with WT‐circ‐POSTN or MUT‐circ‐POSTN and miR‐876‐5p or miR‐NC was determined. (d and e) The levels of miR‐876‐5p and circ‐POSTN in TE‐1 and KYSE410 cells incubated with IgG or Ago2 antibody were detected using RNA immunoprecipitation (RIP) assay. (f and g) The levels of circ‐POSTN and miR‐876‐5p were measured in TE‐1 and KYSE410 cells transfected with sh‐NC, sh‐circ‐POSTN, pcDNA, or circ‐POSTN overexpression vector. (h) The abundance of miR‐876‐5p was determined in normal tissues (*n* = 30) and EC tissues (*n* = 30). (I) The expression of miR‐876‐5p was examined in human esophageal epithelial cell line (HEEC) cells and EC cells (TE‐1 and KYSE410). (j) Correlation between circ‐POSTN and miR‐876‐5p expression in 30 cases of EC tissues was determined by Spearman's rank correlation coefficient. *p < 0.05.

CircRNAs can function as sponges for miRNAs and modulate miRNA expression.^21^ We searched for miRNAs with the binding site of circ‐POSTN by utilizing the online bioinformatic database (starBase version 2.0). As shown in Figure 3a, miR‐876‐5p was predicted as a target for circ‐POSTN. To identify whether miR‐876‐5p was targeted by circ‐POSTN, dual‐luciferase reporter and RIP assays were conducted. Overexpression of miR‐876‐5p inhibited the luciferase activity of the WT‐circ‐POSTN group but not the MUT‐circ‐POSTN group (Figure 3b,c). In addition, the enrichments of circ‐POSTN and miR‐876‐5p were notably increased in the Ago2 group with respect to the IgG group (Figure 3d,e). Furthermore, the transfection efficiencies of sh‐circ‐POSTN and circ‐POSTN overexpression vector were detected, and the results disclosed that circ‐POSTN level was reduced by transfection of sh‐circ‐POSTN, whereas it was increased by transfection of circ‐POSTN overexpression vector (Figure 3f). Moreover, the silencing of circ‐POSTN led to a promotion in miR‐876‐5p level, while the abundance of miR‐876‐5p was decreased by circ‐POSTN overexpression in TE‐1 and KYSE410 cells (Figure 3g). Additionally, miR‐876‐5p abundance in EC tissues and cells was further analyzed. As shown in Figure 3h, miR‐876‐5p abundance was decreased in EC tissue samples. Likewise, the miR‐876‐5p level was lower in TE‐1 and KYSE410 cells than that in HEEC cells (Figure 3i). In addition, there was an inverse correlation between miR‐876‐5p expression and circ‐POSTN level in EC tissue samples (Figure 3j). Taken together, circ‐POSTN functioned as a sponge for miR‐876‐5p.

### Knockdown of miR‐876‐5p abolished the impact of circ‐POSTN silence on proliferation, apoptosis, invasion, and radiosensitivity in EC cells

**FIGURE 4:**
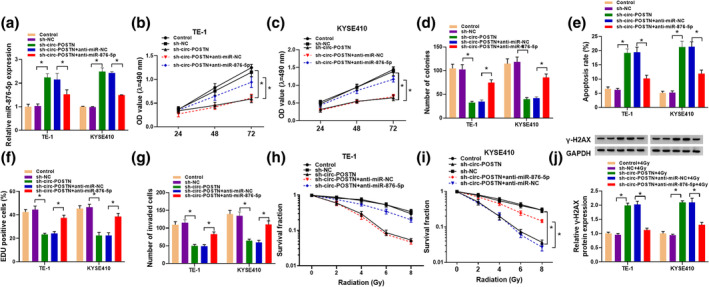
Circ‐POSTN silence regulated cell proliferation, apoptosis, invasion and radiosensitivity of esophageal cancer (EC) cells by sponging miR‐876‐5p. TE‐1 and KYSE410 cells were divided into 5 groups: Control (untreated cells), sh‐NC, sh‐circ‐POSTN, sh‐circ‐POSTN + anti‐miR‐NC, or sh‐circ‐POSTN + anti‐miR‐876‐5p. (a) The abundance of miR‐876‐5p was evaluated via reverse transcription‐quantitative polymerase chain reaction (RT‐qPCR) analysis. (b–d) Cell viability and colony‐formation ability were detected by 3‐(4,5‐dimethylthiazol‐2‐yl)‐2,5‐diphenyl‐2H‐tetrazolium bromide (MTT) assay and colony formation assay. (e) Cell apoptosis was examined by flow cytometry assay. (f) DNA synthesis was measured by 5‐ethynyl‐2′‐deoxyuridine (EdU) staining assay. (g) Number of invaded cells was analyzed by transwell assay. (h and i) Cell survival fraction in TE‐1 and KYSE410 cells exposed to different doses of radiation was measured by colony formation assay. (j) The protein level of γ‐H2AX was detected by western blot in TE‐1 and KYSE410 cells exposed to 4 Gy radiation *p < 0.05.

Subsequently, we further investigated the function of miR‐876‐5p in circ‐POSTN‐mediated EC cell progression. Circ‐POSTN silence elevated miR‐876‐5p level, while this effect was abated by inhibition of miR‐876‐5p (Figure 4a). Additionally, the inhibiting effects of sh‐circ‐POSTN on cell viability and cell colony formation were reversed by miR‐876‐5p knockdown in TE‐1 and KYSE410 cells (Figure 4b–d). In addition, the proapoptosis, antiproliferation, and anti‐invasion effects caused by circ‐POSTN silence were overturned by miR‐876‐5p downregulation (Figure 4e–g). Moreover, we found that suppression of miR‐876‐5p also attenuated the effects of sh‐circ‐POSTN on survival fraction and γ‐H2AX protein level in TE‐1 and KYSE410 cells exposed to radiation (Figure 4h–j). Collectively, circ‐POSTN exerted its roles in EC cells via sponging miR‐876‐5p.

### MiR‐876‐5p directly targeted FYN

**FIGURE 5:**
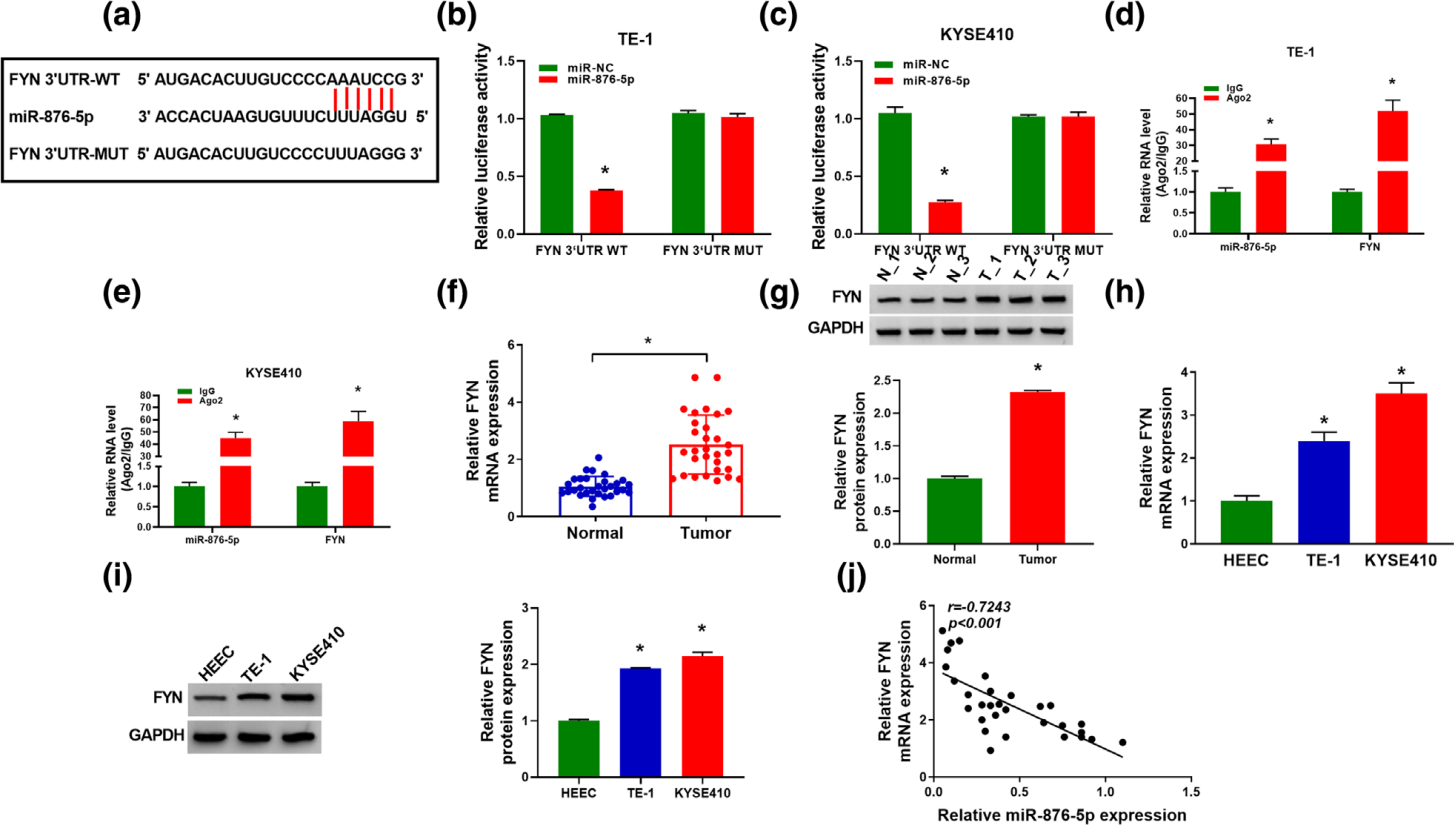
Proto‐oncogene tyrosine‐protein kinase (FYN) was targeted by miR‐876‐5p. (a) The putative binding sites between miR‐876‐5p and FYN 3'UTR were predicted by starBase version 2.0. (b and c) The interaction between FYN and miR‐876‐5p was verified by dual‐luciferase reporter. (d and e) The levels of miR‐876‐5p and FYN in TE‐1 and KYSE410 cells incubated with IgG or Ago2 antibody were analyzed by RNA immunoprecipitation (RIP) assay. (f and g) Reverse transcription‐quantitative polymerase chain reaction (RT‐qPCR) and western blot assays were performed to analyze the mRNA and protein levels of FYN in 30 pairs of normal tissues and EC tissues. (h and i) FYN mRNA and protein levels were detected in human esophageal epithelial cell line (HEEC) cells and EC cells (TE‐1 and KYSE410). (j) Correlation between FYN and miR‐876‐5p expression in 30 cases of EC tissues was analyzed by Spearman's rank correlation coefficient. *p < 0.05.

Subsequently, we used the online software starBase version 2.0 to predict the potential targets of miR‐876‐5p, and FYN was predicted to be a target for miR‐876‐5p (Figure 5a). As described in Figure 5b,c, miR‐876‐5p elevation apparently suppressed the luciferase activity of FYN 3'UTR‐WT, but the luciferase activity of FYN 3'UTR‐MUT did not change significantly. In addition, miR‐876‐5p and FYN were greatly enriched in Ago2‐containing beads relative to the IgG control group (Figure 5d,e). FYN mRNA and protein levels were increased in EC tissues (Figure 5f,g). Moreover, FYN mRNA and protein levels were enhanced in EC cells (TE‐1 and KYSE410) compared to HEEC cells (Figure 5h,i). In addition, the expression of FYN was inversely correlated with miR‐876‐5p abundance in EC tissues (Figure 5j). Hence, FYN was targeted by miR‐876‐5p.

### Overexpression of FYN attenuated the influence of miR‐876‐5p upregulation on proliferation, apoptosis invasion, and radiosensitivity in EC cells

**FIGURE 6:**
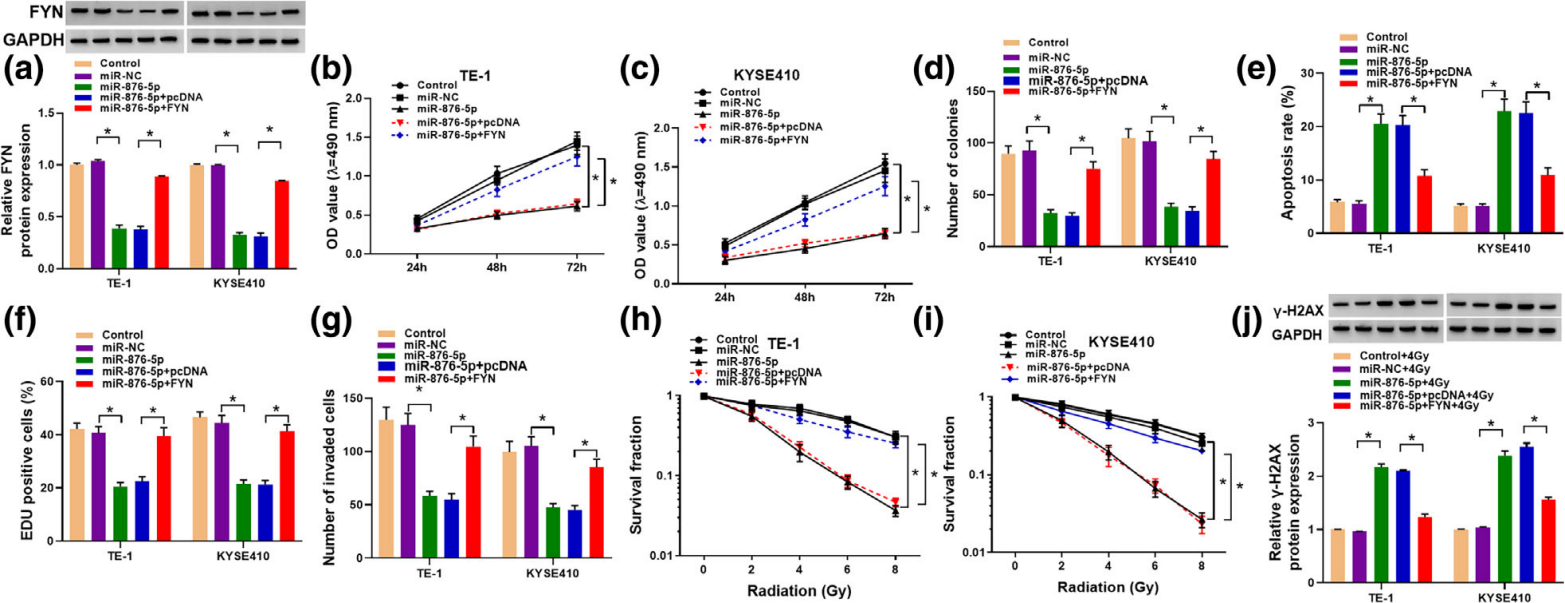
Overexpression of miR‐876‐5p regulated the progression of esophageal cancer (EC) by regulating proto‐oncogene tyrosine‐protein kinase (FYN) expression. TE‐1 and KYSE410 cells were divided into five groups: Control (untreated cells), miR‐NC, miR‐876‐5p, miR‐876‐5p + pcDNA, and miR‐876‐5p + FYN. (a) Western blot assay was employed to examining the protein expression of FYN. (b–d) Cell viability and cell colony‐forming ability were evaluated by 3‐(4,5‐dimethylthiazol‐2‐yl)‐2,5‐diphenyl‐2H‐tetrazolium bromide (MTT) and colony formation assays. (e) Flow cytometry analysis was used to examine cell apoptosis. (f) DNA synthesis was determined by 5‐ethynyl‐2′‐deoxyuridine (EdU) assay. (g) Cell invasion was assessed by transwell assay. (h and i) Colony formation assay was employed to examine survival fraction in TE‐1 and KYSE410 cells exposed to radiation. (j) The protein expression of γ‐H2AX in TE‐1 and KYSE410 cells exposed to 4 Gy radiation was determined by Western blot assay. *p < 0.05.

To determine whether FYN mediated the biological role of miR‐876‐5p, rescue experiments were carried out. WB assay revealed that elevation of miR‐876‐5p led to a marked repression of FYN protein expression, while overexpression of FYN abolished this effect (Figure 6a). The functional experiments disclosed that miR‐876‐5p overexpression reduced cell viability, colony formation, EDU‐positive cells, and invasion and increased apoptosis in TE‐1 and KYSE410 cells, which were abated by the addition of FYN (Figure 6b–g). In addition, miR‐876‐5p upregulation drastically suppressed the survival fraction and increased γ‐H2AX protein expression in TE‐1 and KYSE410 cells treated with radiation, while these effects were reversed by upregulating FYN (Figure 6h–j). Thus, miR‐876‐5p restoration impeded cell progression and enhanced radiosensitivity in EC by downregulating FYN.

### Circ‐POSTN regulated FYN expression via sponging miR‐876‐5p in EC cells

**FIGURE 7:**
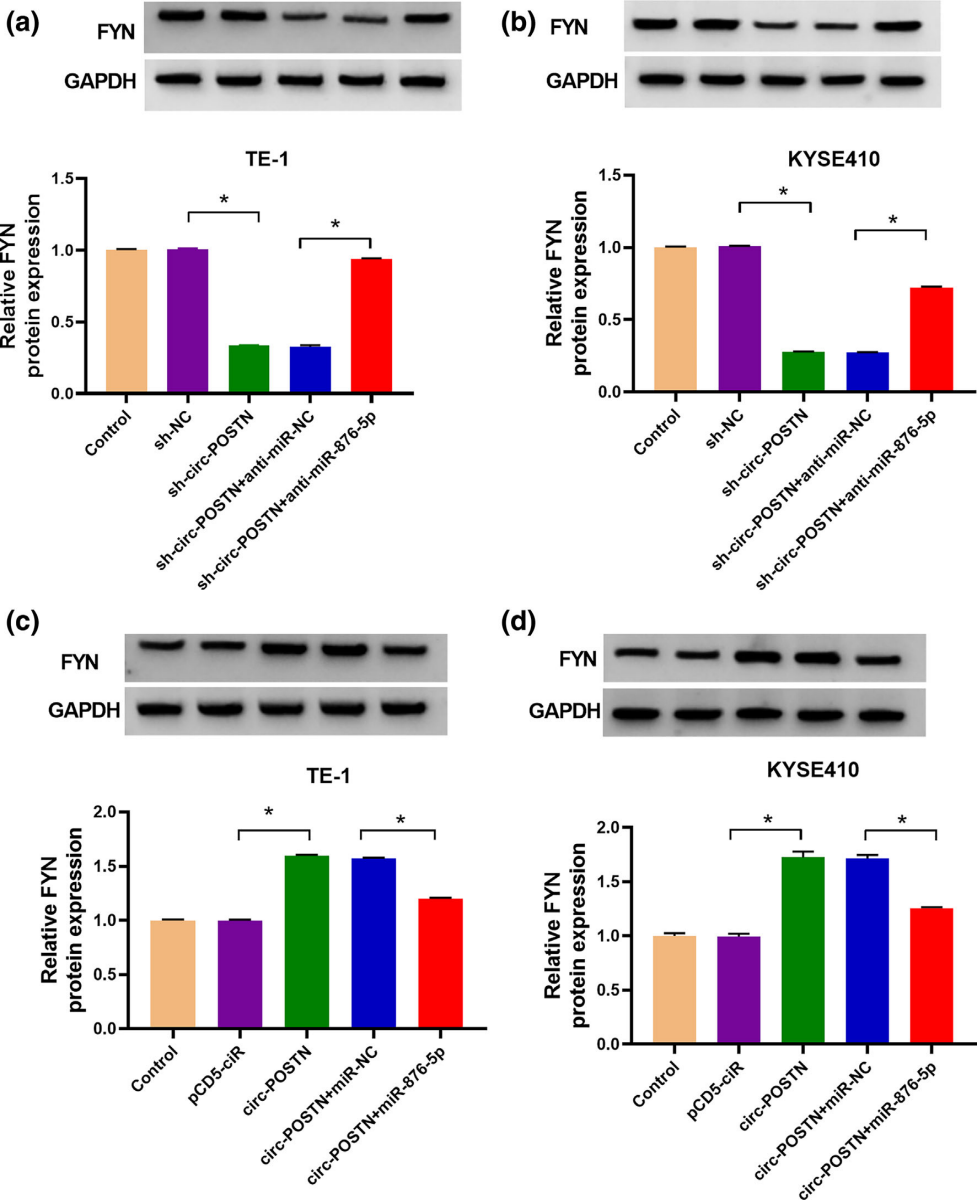
Circ‐POSTN regulated the expression of proto‐oncogene tyrosine‐protein kinase (FYN) by sponging miR‐876‐5p in esophageal cancer (EC) cells. (a and b) Western blot assay was applied to determine FYN protein level in TE‐1 and KYSE410 cells transfected with sh‐NC, sh‐circ‐POSTN, sh‐circ‐POSTN + anti‐miR‐NC, or sh‐circ‐POSTN + anti‐miR‐876‐5p, and untreated cells were used as control group. (c and d) The protein expression of proto‐oncogene tyrosine‐protein kinase (FYN_ was detected by Western blot assay in TE‐1 and KYSE410 cells transfected with pcDNA, circ‐POSTN, circ‐POSTN + miR‐NC, or circ‐POSTN + miR‐876‐5p, and untreated cells were used as control group. *p < 0.05.

Since miR‐876‐5p was inversely regulated by circ‐POSTN, we wondered whether circ‐POSTN could function as a miR‐876‐5p sponge to affect FYN level. Western blot analysis uncovered that the knockdown of circ‐POSTN decreased the protein level of FYN, which was restored by the downregulation of miR‐876‐5p (Figure 7a,b). Moreover, FYN protein level was increased by overexpression of circ‐POSTN, which was reversed by upregulating miR‐876‐5p (Figure 7c,d), indicating that circ‐POSTN regulated FYN through serving as a sponge of miR‐876‐5p.

### Silencing of circ‐POSTN limited tumor growth and enhanced radiosensitivity in EC in vivo

**FIGURE 8:**
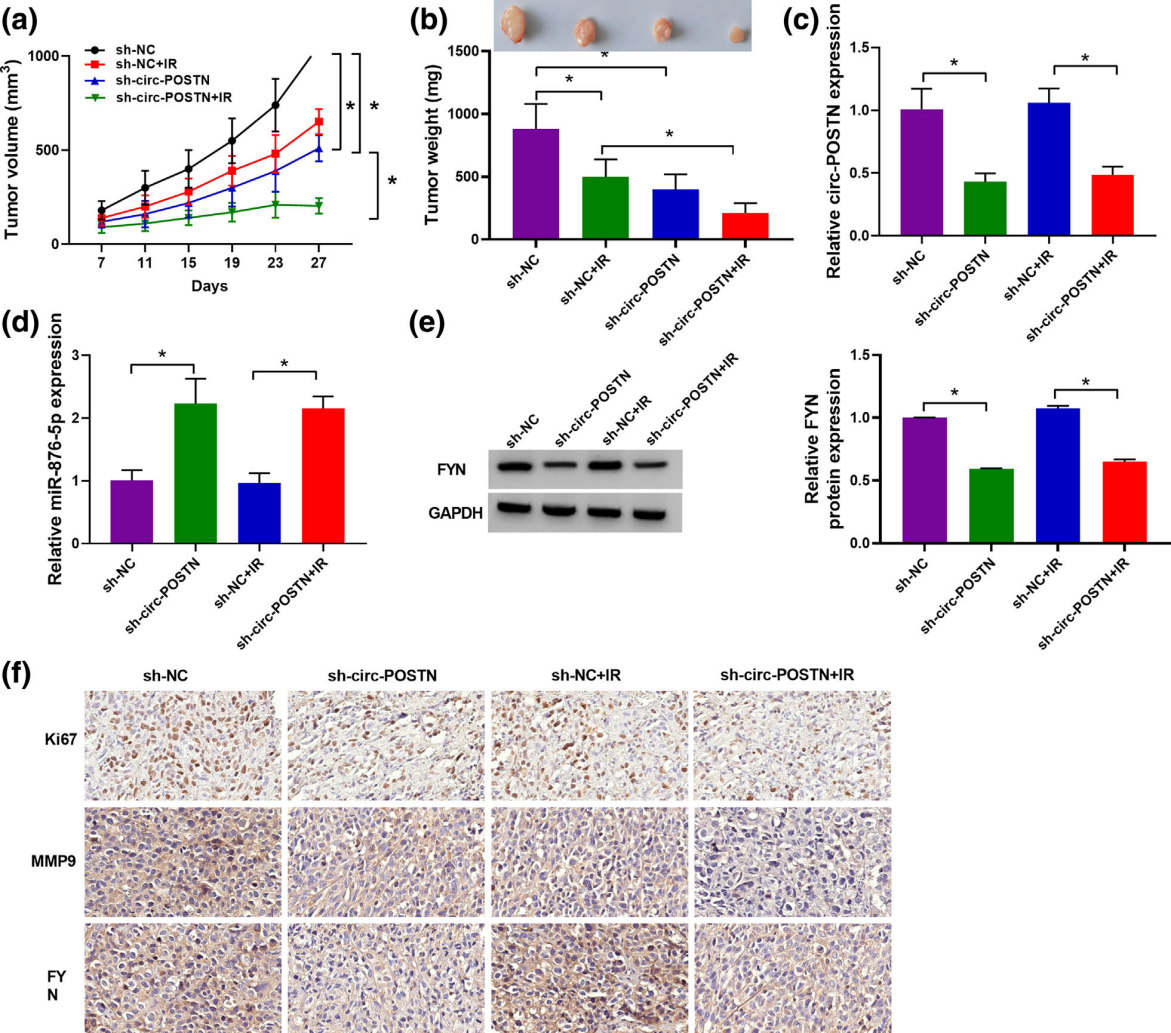
Silencing of circ‐POSTN inhibited tumor growth and enhanced radiosensitivity of EC cells in vivo. KYSE410 cells stably transfected with sh‐circ‐POSTN or sh‐NC (as control) were injected into nude mice for 7 days, and then irradiated with or without 6 Gy x‐ray. (a and b) Tumor volume and weight were measured. (c–e) The expression levels of circ‐POSTN, miR‐876‐5p, and proto‐oncogene tyrosine‐protein kinase (FYN) protein in resected tumor tissues were examined. (f) The expression levels of Ki67, MMP9 and FYN in xenograft tumor tissues were determined by immunohistochemistry (IHC) analysis. *p < 0.05.

To determine the effects of circ‐POSTN knockdown on tumor growth and radiosensitivity in vivo, sh‐circ‐POSTN or sh‐NC‐transfected KYSE410 cells were subcutaneously injected into nude mice, followed by irradiation with or without 6 Gy x‐ray every 4 days after injection for 7 days. In agreement with in vitro data, tumor volume and weight were inhibited by IR treatment or circ‐POSTN knockdown, while this effect was significantly enhanced in mice with circ‐POSTN silence and IR treatment (Figure 8a,b). In addition, transfection of sh‐circ‐POSTN reduced circ‐POSTN expression and FYN protein level, but increased miR‐876‐5p expression in tumor tissues of the sh‐circ‐POSTN group and sh‐circ‐POSTN+IR group (Figure 8c–e). IHC analysis showed that positive cells of Ki‐67 and MMP9 were decreased in mice with sh‐circ‐POSTN transfection or IR treatment, and this tendency was further enhanced by the combination of sh‐circ‐POSTN transfection and the IR treatment (Figure 8f). However, FYN‐positive cells were reduced in cells with sh‐circ‐POSTN transfection or sh‐circ‐POSTN transfection and IR treatment but had little change in the IR treatment group (Figure 8f). Altogether, circ‐POSTN silence limited tumor growth and enhanced radiosensitivity in EC cells by regulating the miR‐876‐5p/FYN axis in vivo.

## DISCUSSION

An increasing body of evidence has suggested that dysregulation of circRNAs participates in diverse biological processes and radiosensitivity of cancers.^22^, ^23^ However, few studies have focused on the functions of aberrantly expressed circRNAs in modulating the radiosensitivity of EC. In this study, we studied the biological role of circ‐POSTN in proliferation, apoptosis, invasion, and radiosensitivity of EC cells.

It has previously been reported that circ‐POSTN was overexpressed in glioma tissue specimens and cell lines, and accelerated cell proliferation and invasion through downregulating miR‐433‐3p.^24^ In addition, previous research disclosed that circ‐POSTN was also highly expressed in EC cells, and its silence limited cell growth, migration, and EMT process via miR‐599/ENAH axis.^10^ However, there have been no studies on the effects of circ‐POSTN on the radiosensitivity of cancers. In our study, a high level of circ‐POSTN was observed in EC tissue specimens and cell lines, and circ‐POSTN depletion could repress cell proliferation and invasion, but promoted apoptosis and enhanced radiosensitivity in EC cells in vitro. In addition, circ‐POSTN silence curbed tumor growth and strengthened radiosensitivity in EC in vivo. Altogether, circ‐POSTN silence restrained tumorigenesis and increased radiosensitivity of EC in vitro and in vivo.

It is widely accepted that circRNAs can interact with miRNAs via functioning as competing endogenous RNAs, and the circRNA‐miRNA axis is involved in many of the biological behaviors of cells, including cell growth, apoptosis, and metastasis.^21^, ^25^ Therefore, we hypothesized that circ‐POSTN exerted its functions through interacting with miRNAs. miR‐876‐5p was predicted to contain the complementary binding fragments of circ‐POSTN. MiR‐876‐5p has been indicated to play a tumor‐suppressing role in several forms of cancer. For instance, Zhao et al. revealed that miR‐876‐5p restrained proliferation, migration, invasion and stemness of non‐small cell lung cancer via the downregulating WNT5A.^26^ Additionally, miR‐876‐5p repressed the aggressiveness of oral squamous cell carcinoma through downregulation of zeste homolog‐2 (EZH2).^27^ Notably, previous research illustrated that miR‐876‐5p level declined in EC cells, and its upregulation inhibited EC progression.^8^ Consistent with previous studies, miR‐876‐5p was lowly expressed in EC tissues and EC cells. In addition, miR‐876‐5p interference could abolish the influences of circ‐POSTN interference on cell proliferation, invasion, apoptosis, and radiosensitivity in EC cells. Altogether, these results suggested that circ‐POSTN knockdown inhibited EC cell progression and enhanced radiosensitivity via sponging miR‐876‐5p. In addition, circ‐POSTN has been reported to promote the proliferation of EC cells and human glioma cells via sponging multiple miRNAs.^10^, ^24^ These findings indicate that circ‐POSTN is involved in the complex regulatory networks and confers cell‐type‐specific regulation of cell function in different cancers.

Given that miRNAs exert their functions via targeting specific genes, the possible targets of miR‐876‐5p were explored. We confirmed that FYN was targeted by miR‐876‐5p. FYN plays an essential role in diverse cellular processes.^28^, ^29^ In addition, previous studies showed that FYN served as a tumor accelerator in multiple cancers.^17^, ^30^, ^31^ Moreover, Liu et al. disclosed that FYN expression was elevated in EC tissue specimens, and FYN silence restrained cell growth and facilitated apoptosis in EC cells.^19^, ^20^ Here, FYN was also observed to be overexpressed in EC tissue specimens and EC cell lines. We uncovered that FYN enhancement weakened the influences of miR‐876‐5p restoration on cell progression and radiosensitivity in EC cells. In addition, circ‐POSTN could upregulate FYN expression through sponging miR‐876‐5p. Additionally, we found that FYN expression in xenograft tumor tissues that were exposed to IR treatment had no significant change compared to the sh‐NC group, while it was obviously decreased in xenograft tumor tissues with sh‐circ‐POSTN treatment. Hence, circ‐POSTN could regulate EC cell progression and radiosensitivity by miR‐876‐5p/FYN axis in vitro and in vivo. Our study enriched the understanding of the molecular mechanism by which circ‐POSTN affects EC genesis. Compared with previous studies, the novelty of this study is that circ‐POSTN knockdown might repress EC tumor growth and improve radiosensitivity in vivo.

**FIGURE 9:**
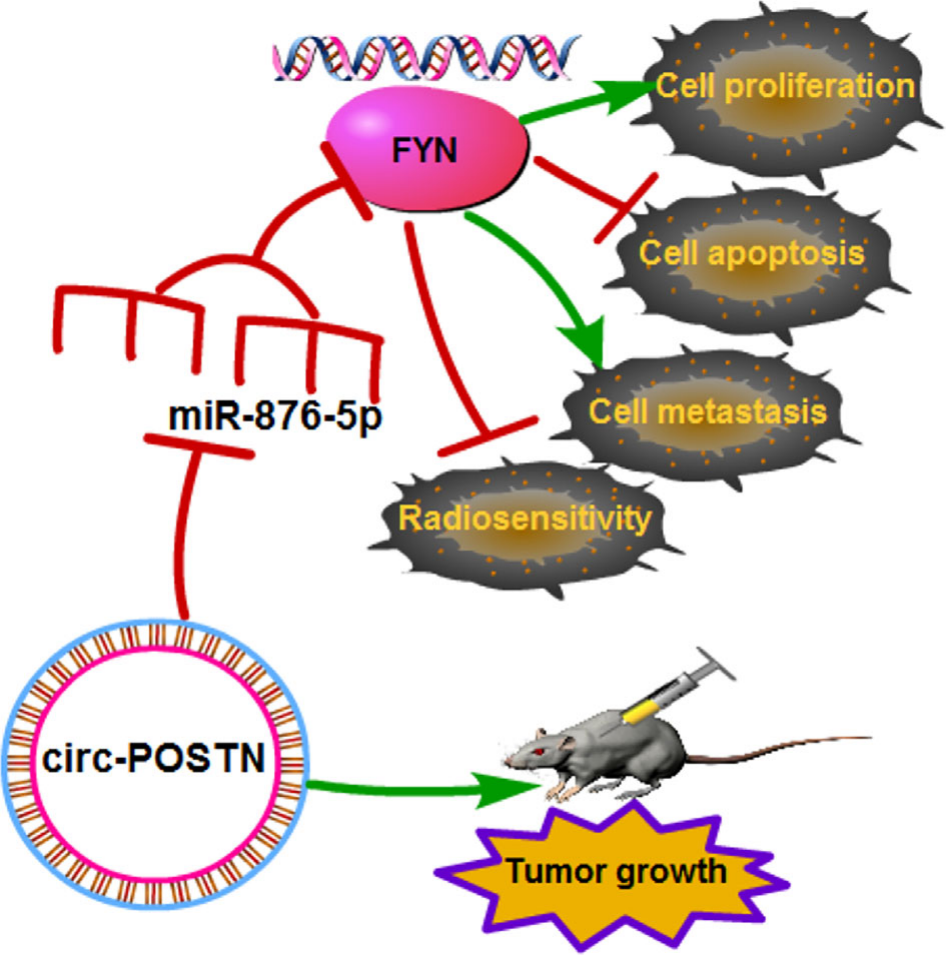
Schematic diagram of circ‐POSTN/miR‐876‐5p/FYN axis in regulating cell proliferation, apoptosis, metastasis, and radiosensitivity in esophageal cancer (EC) cells.

In conclusion, circ‐POSTN and FYN were overexpressed, but miR‐876‐5p was lowly expressed in EC tissues and cells. Circ‐POSTN regulated EC cell proliferation, apoptosis, invasion, and radiosensitivity via modulating miR‐876‐5p/FYN axis (Figure 9). Overall, circ‐POSTN might be a novel and effective target for EC treatment.

## AUTHOR CONTRIBUTIONS

Alan Chu, Yongtai Wang and Chen Sun designed and performed the research; Zongwei Liu, Shijia Liu, Mengxi Li and Rui Song analyzed the data; Alan Chu, Lanlan Gan and Ruitai Fan wrote the manuscript. All authors read and approved the final manuscript.

## FUNDING INFORMATION

Joint Construction Project of Medical Science and Technology in Henan Province (LHG20220487).

## CONFLICT OF INTEREST STATEMENT

The authors declare no conflict of interest.
